# Muscone inhibits angiotensin II–induced cardiac hypertrophy through the STAT3, MAPK and TGF-β/SMAD signaling pathways

**DOI:** 10.1007/s11033-023-08916-1

**Published:** 2023-12-29

**Authors:** Yi-jiang Liu, Jia-jia Xu, Cui Yang, Yan-lin Li, Min-wei Chen, Shi-xiao Liu, Xiang-hui Zheng, Ping Luo, Rui Li, Di Xiao, Zhong-gui Shan

**Affiliations:** 1 Present Address: School of Medicine, The First Affiliated Hospital of Xiamen University, Xiamen University, Xiamen, 361003 China; 2 Present Address: The Third Clinical Medical College, Fujian Medical University, Fujian, China

**Keywords:** Muscone, Angiotensin II, Cardiac hypertrophy, Cardiac fibrosis, Inflammation, STAT3, MAPK, TGF-β/SMAD

## Abstract

**Background:**

Muscone is a chemical monomer derived from musk. Although many studies have confirmed the cardioprotective effects of muscone, the effects of muscone on cardiac hypertrophy and its potential mechanisms are unclear.The aim of the present study was to investigate the effect of muscone on angiotensin (Ang) II-induced cardiac hypertrophy.

**Methods and results:**

In the present study, we found for the first time that muscone exerted inhibitory effects on Ang II-induced cardiac hypertrophy and cardiac injury in mice. Cardiac function was analyzed by echocardiography measurement, and the degree of cardiac fibrosis was determined by the quantitative real-time polymerase chain reaction (qRT-PCR), Masson trichrome staining and western blot assay. Secondly, qRT-PCR experiment showed that muscone attenuated cardiac injury by reducing the secretion of pro-inflammatory cytokines and promoting the secretion of anti-inflammatory cytokines. Moreover, western blot analysis found that muscone exerted cardio-protective effects by inhibiting phosphorylation of key proteins in the STAT3, MAPK and TGF-β/SMAD pathways. In addition, CCK-8 and determination of serum biochemical indexes showed that no significant toxicity or side effects of muscone on normal cells and organs.

**Conclusions:**

Muscone could attenuate Ang II-induced cardiac hypertrophy, in part, by inhibiting the STAT3, MAPK, and TGF-β/SMAD signaling pathways.

**Supplementary Information:**

The online version contains supplementary material available at 10.1007/s11033-023-08916-1.

## Introduction

As a pumping organ, the heart’s primary function is to maintain blood perfusion to peripheral organs and thus maintain their functional state. In response to a large number of physiological and pathological stimuli, the myocardium responds with cardiac hypertrophy [1]. Pathological and physiological cardiac hypertrophy occurs when the pumping capacity of the heart is insufficient to meet the demands of peripheral organs. When the preload or afterload of the heart increases, the mass and size of cardiac myocytes increase, and the parallel modular units increase, resulting in increased contractile capacity of the heart [2]. In addition, the development of myocardial hypertrophy is accompanied by qualitative changes, such as changes in gene expression, leading to changes in metabolism, contractility, and cardiomyocyte survival [3]. Pathological myocardial hypertrophy is a major cause of cardiac remodeling and heart failure with high morbidity and mortality, and it affects the quality of life and patient survival [4–6]. Although pathological myocardial hypertrophy involves multiple stimuli, neurohumoral activation is the key factor [7, 8]. Currently, targeting the renin-angiotensin-aldosterone system (RAAS) is a recognized clinical approach independent of blood pressure to reverse maladaptive cardiac hypertrophy [3].

Angiotensin II (Ang II) is a biologically active octapeptide in the RAAS. Ang II is a major effector of the RAAS, and elevated levels of Ang-II in vivo lead to increased expression of genes related to hypertension and many marker proteins associated with late stage cardiac hypertrophy [3]. Ang II also induces ROS production in cardiomyocytes. Persistent myocardial hypertrophy leads to cardiomyocyte death, resulting in cardiac dysfunction and even chronic heart failure [9]. Angiotensin II induces cardiac hypertrophy in vivo and in vitro, mainly mediated through the angiotensin II type 1 receptor (AT1R). Animal models of cardiac-specific overexpression of AT1R show enhanced hypertrophy leading to heart failure due to pressure overload [10]. However, cardiac AT1R knockdown results in improved cardiac systolic function and attenuated angiotensin-converting enzyme 2 (ANG II)-mediated hypertrophic response in patients with myocardial infarction [11]. Myocardial hypertrophy involves many signaling pathways, such as the JAK2/STAT3, MAPK, TGF-β/SMAD, and RAC-α/Akt signaling pathways [1, 12, 13]. More importantly, the MAPK pathway is activated by the AT1R and is involved in pathological hypertrophic responses. Although, there has been many studies on the pathophysiological mechanisms underlying mammalian cardiac hypertrophy, there is no effective treatment for myocardial hypertrophy in the clinical setting. Therefore, the search for new therapeutic approaches for the treatment of myocardial hypertrophy is crucial to provide a viable treatment for the reversal of heart failure.

The chemical structure of muscone is 3-methylcyclopentadecanone, which is the main active component of musk [14]. Previous studies have suggested that musk ketones are an antidote to poisoning [15, 16]. Muscone has been investigated in many studies due to its various potential effects, such as antitumor, antidementia, antidiabetic peripheral neuropathy, and anti-ischemia heart disease effects [14]. However, most of the pharmacological mechanisms of action of muscone remain unclear. Although many studies have confirmed the cardioprotective effects of muscone [17–20], the effects of muscone on myocardial hypertrophy and its potential mechanisms are unclear. Therefore, the aim of the present study was to design an animal model of myocardial hypertrophy and to observe the effect of muscone on ANG II-induced myocardial hypertrophy.

## Materials and methods

### Animals and treatments

Six-week male C57BL/6 mice, weighing approximately 22–25 g, were purchased from Beijing Vital River Laboratory Animal Technology Co. Mice were kept in a specific-pathogen-free (SPF) environment, which was maintained at a constant temperature and humidity, and they were given sufficient water and feed. ALZET® Osmotic Pumps (2004 model, USA) were immersed in saline for 8 h in advance, and Ang II (soluble in saline; Cat. No. A9525, Sigma-Aldrich, USA) was dissolved in normal saline and injected into the osmotic pumps. A transverse incision (1 cm) was made in the middle of the back of the mice, and the osmotic pump was implanted under the skin. The infusion rate of Ang II was 0.25 µL/h, and the concentration was 1.5 µg/kg per min. The mice were randomly divided into six groups with six mice in each group as follows: saline group (sham); saline + 2 mg/kg/d muscone (Cat. No. HY-N0633, MedChemExpress Technology, China) group; Ang II group; 2 mg/kg/d muscone + Ang II group; 4 mg/kg/d muscone + Ang II group; and 4 mg/kg valsartan group (positive control; Cat. No. HY-18,204, MedChemExpress Technology, China). Muscone was dissolved in normal saline, and valsartan was dissolved in 0.5% carboxylmethyl cellulose sodium (CMC-Na) solution. The mice were given 200 µL of muscone or valsartan via intragastric gavage once every day. The sham and Ang II groups received an equivalent volume of solvent. Tissues were sampled after 28 days.

### Echocardiography measurement

Mice were anesthetized with 1.5% isoflurane, and their anterior chest hair was removed. After smearing the ultrasound coupling agent on their chest, echocardiographic parameters were obtained by a VisualSonics high-resolution Vevo 2100 system (VisualSonics, Toronto, Canada).

### Histopathological studies

The fixed cardiac tissues were embedded in paraffin and then cut into 5 μm thick sections. H&E staining was used to assess myocardial structural. A Masson’s trichrome staining kit (Cat. No. D026-1-3, Nanjing Jiancheng, China) was used to assess the degree of fibrosis. Photomicrographs were taken at 400x magnification by an intelligent biological microscope (OLYMPUS BX53). The percentage of collagen-stained area (blue) was calculated using a quantitative digital image analysis system (Image-Pro Plus 6.0 software).

### Target forecasting, enrichment analysis, and AutoDock molecular docking studies

The structure of muscone was downloaded from ChemSpider (www.chemspider.com, ID 10,483) for prediction of protein targets via SwissADME (http://www.swissadme.ch/). We enriched the predicted target proteins by R language analysis. The crystal structures of proteins (SMAD2, SMAD3, SMAD4, SMAD7, ERK, P38, and JNK) were extracted from the Research Collaboratory for Structural Bioinformatics (RCSB) Protein Data Bank (PDB) (https://www.rcsb.org/) in complex with muscone and used as docking structure templates in AutoDockTools (1.5.7). Molecular docking studies were performed using the Glide tool in the Schrödinger Maestro suite. The proteins were processed using the protein preparation wizard to ensure chemical correctness and to optimize the protein structure for docking.

### Quantitative real-time polymerase chain reaction (qRT-PCR)

Total RNA was isolated from mouse heart tissues using TRIzol reagent (Takara, Japan) and reverse transcribed into cDNA with the Takara Reverse transcription kit and PrimeScript RT Master Mix (Cat. No. RR036A). The mRNA levels of genes were quantitatively examined using a fluorescence quantitative PCR instrument (Roche LightCycler 480) with Fast SYBR™ Green Master Mix (Cat. No. 4,385,610, Thermo Fisher, Waltham, MA, USA). The primer sequences are listed in Supplementary Table S1. The relative expression of each gene was calculated with the 2-^ΔΔCt^ method.

### Western blot

The heart tissues and H9C2 cells were lysed with RIPA lysis buffer (Beyotime Institute of Biotechnology, Shanghai, China), and protein concentrations were determined using a bicinchoninic acid (BCA) protein kit (Beyotime, China). The proteins were separated with 8% or 12% sodium dodecyl sulfate polyacrylamide gel electrophoresis (SDS-PAGE) and then transferred to a polyvinylidene fluoride (PVDF) membrane (Beyotime, China). The PVDF membrane was blocked with 5% skim milk for 1 h and then incubated with the following primary antibodies at 4 °C overnight: GAPDH (1:10,000, Cat. No. 60004-1-Ig, Proteintech), ANP (1:1,000, Cat. No. 27426-1-AP, Proteintech), α-SMA (1:1,000, Cat. No. 14395-1-AP, Proteintech), COL1A1 (1:1,000, Cat. No. 72026T, Cell Signaling Technology), β-MHC (1:1,000, Cat. No. ab-50,967, Abcam), STAT3 (1:1,000, Cat. No. 9139 S, Cell Signaling Technology), p-STAT3 (1:1,000, Cat. No. 9145 S, Cell Signaling Technology), ERK (1:1,000, Cat. No. 4695 S, Cell Signaling Technology), p-ERK (1:1,000, Cat. No. 4370 S, Cell Signaling Technology), p-P38 (1:1,000, Cat. No. 8242 S, Cell Signaling Technology), P38 (1:1,000, Cat. No. 8242 S, Cell Signaling Technology), SMAD3 (1:1,000, Cat. No. A19115, Abclonal Technology), p-SMAD3 (1:1000, Cat. No. 380,775, ZEN-BIOSCIENCE, China), p-SMAD2 (1:1,000, Cat. No. AP0269, Abclonal Technology), and SMAD2 (1:1000, Cat. No. 200,790, Zen Bioscience, China). After washing the membrane three times with tris-buffered saline with Tween 20 (TBST), it was incubated with horseradish peroxidase-conjugated goat anti-rabbit or goat anti-mouse secondary antibodies. The protein bands were detected by an enhanced chemiluminescence (ECL) detection kit (Millipore, USA). An antibody diluent (Cat. No. W019-1-1, Nanjing Jiancheng, Nanjing, China) was used to dilute the primary and secondary antibodies as indicated above.

### Cell culture

H9C2 rat embryonic cardiomyocytes were purchased from Procell Life Science & Technology Company (Wuhan, China). Human AC16 cardiomyocytes were obtained from ATCC (Manassas, VA, USA). H9C2 cells and AC16 cells were cultured with high-glucose Dulbecco’s modified Eagle’s medium (DMEM) supplemented with 10% fetal bovine serum (FBS) (Cat. No. Z7186FBS, Zeta-Life, France), penicillin (100 U/mL), and streptomycin (100 µg/ml). Cell lines were cultured in a humidified incubator with 5% CO_2_ at 37 °C.

### Cell viability assay

Cell viability was assessed by a Cell Counting Kit-8 (CCK-8) assay (Cat. No. K1018, APExBIO Technology, USA). H9C2 and AC16 cells were seeded into 96-well plates at 1 × 10^4^ cells per well. After 24 h of incubation, cells were treated with muscone (purity > 98%, Cat. No. HY-N0633, MedChemExpress Technology, USA) at serial concentrations (0, 0.125, 0.5, 1.0, 2.0, 4.0, 8.0, and 16 µg/ml) for 24 h. Then, 10 µL of CCK-8 reagent was added to each well, and the absorbance at 450 nm was measured using a microplate reader to evaluate cell viability.

### Determination of serum biochemical indexes

The following biochemical indexes were examined in the serum samples collected from the experimental mice: a heart function index, namely, lactate dehydrogenase (LDH); liver function indexes, namely, alanine aminotransferase (ALT) and aspartate aminotransferase (AST); and kidney function indexes, namely, urea formaldehyde (UF) and creatinine sox (CREA-S). All indexes were detected by an automatic chemistry analyzer (BS-240vet, Mindray Bio-Medical Electronics Co.)

### Statistical analysis

All results are presented as the mean ± standard deviation (SD) and analyzed using GraphPad (version 8.0.1, GraphPad Prism Software). Differences of each sample were evaluated using an unpaired Student’s two-tailed *t*-test or analysis of variance (ANOVA). A value of p < 0.05 was considered significant. All experiments were repeated at least three times.

## Results

### Muscone reduces Ang II-induced myocardial hypertrophy

**Fig. 1:**
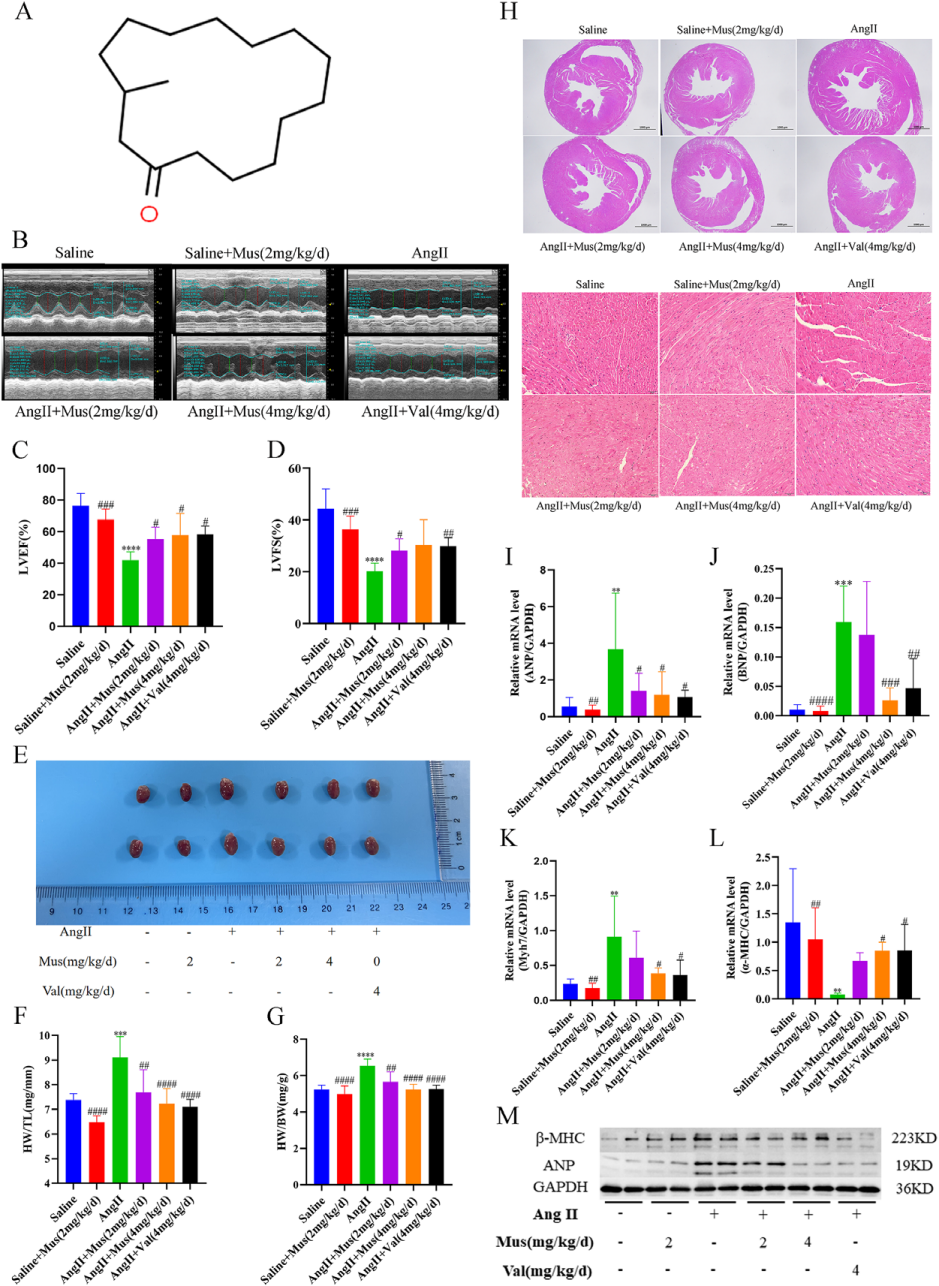
Muscone reduces angiotensin II-induced myocardial hypertrophy. (**A**) The molecular make-up of muscone. (**B**) Exemplary echocardiography images. Echocardiography M-mode imaging from C57BL/6 mice following various therapies. To evaluate heart function, M-mode pictures from short-axis measurements were employed. The diameters of the left ventricle at the diastolic or systolic phases are indicated by vertical arrows. (**C**, **D**) Results of statistical analysis for the left ventricular fractional shortening (LVFS) and left ventricular ejection fraction (LVEF) (LVFS). (**E**) Morphological images of mouse hearts from the saline group, saline + 2 mg/kg muscone group, Ang II group, 2 mg/kg/d muscone + Ang II group, 4 mg/kg/d muscone + Ang II group, and 4 mg/kg valsartan + Ang II group. (**F**, **G**) Ratio of heart weight (HW) to tibia length (TL) and ratio of HW to body weight (BW) in each group. (**H**) Paraffin sections of cardiac tissues were stained with H&E. Representative images are shown. For the first six images, scale bar = 1000 μm. The last six images are the corresponding 400x views of the first six images (scale bar = 50 μm). (**I**-**L**) The mRNA expression levels of ANP, BNP, Myh7 and α-MHC, were measured by qRT-PCR and normalized to GAPDH expression levels. (M) ANP and -MHC protein levels in mouse cardiac tissues as shown by Western blot analysis. We utilized GAPDH as a loading control. Data are presented as the mean ± SD (n = 3–6). *p < 0.05, **p < 0.01, ***p < 0.001, and ****p < 0.0001 vs. the saline group; #p < 0.05, ##p < 0.01, ###p < 0.001, and ####p < 0.0001 vs. the Ang II group

The molecular structure of muscone is shown in Fig. 1A. To investigate the effect of muscone on ANG II-induced myocardial hypertrophy in mice, we used M-mode echocardiography to measure in vivo cardiac function of the six groups of mice (Fig. 1B). The results showed a significant increase in left ventricular systolic internal diameter (LVID) and left ventricular mass (LVM) as well as a decrease in left ventricular ejection fraction (LVEF) and left ventricular fractional shortening (LVFS) in the Ang II group compared to the saline group. However, compared to the Ang II group, the LVEF and LVFS were increased to near normal levels in the muscone treatment group, while the LVID and LVM were significantly decreased to normal levels in the muscone treatment group. Although there was no statistical difference in the LVFS values in the high-dose muscone treatment group (p = 0.0591), there was a subsequent increase in LVEF and LVFS values as well as a decrease in LVID values as the muscone treatment dose increased, but further increases in muscone concentrations did not result in further decreases in LVM (Fig. 1C, D, Supplementary Figure S1A, B). In addition, the left ventricular internal diastolic dimension (LVIDd), interventricular septal thickness at diastole (IVSd), interventricular septal thickness at systolic (IVS), and heart rate (HR) did not differ significantly among the groups (p > 0.05) (Supplementary Figure S1C-F). We also examined the anatomical parameters associated with the Ang II-induced cardiac hypertrophy model in mice to further investigate the effects of muscone on the mouse heart. The morphology of the heart showed that the heart was significantly enlarged after Ang II induction but that the heart in the muscone group was significantly smaller (Fig. 1E). We also measured the body weight (BW), heart weight (HW), and tibial length (TL) in mice and used the ratio of HW to BW as well as the ratio of HW to TL as indicators of myocardial hypertrophy. In Ang II-induced myocardial hypertrophy mice, muscone treatment significantly reduced the HW/BW ratio (mg/g) and the HW/TL ratio (mg/mm) (Fig. 1F, G). In addition, H&E staining of tissue sections further confirmed the inhibitory effect of muscone on Ang II-induced cardiac hypertrophy in C57BL/6 mice (Fig. 1H).

Subsequently, we examined the expression of genes associated with, including atrial natriuretic peptide (ANP), brain natriuretic peptide (BNP), α- myosin heavy chain (α-MHC) and myosin heavy chain 7 (Myh7/β-MHC). qRT-PCR analysis indicated that Ang II significantly increased the mRNA levels of ANP, BNP and Myh7, but these increases were significantly inhibited by muscone treatment, especially at high doses. (Fig. 1I-K). But the results of α-MHC were the opposite (Fig. 1L). In addition, similar results were obtained when protein expression levels of ANP and β-MHC were examined by Western blot analysis. Protein expression of the hypertrophy markers, ANP and β-MHC, was significantly higher in the Ang II-stimulated group compared to the saline group, while the expression of ANP and β-MHC was significantly lower in the muscone treatment group (Figure M, Supplementary Figure S1G, H).

Thus, these findings indicated that muscone has a protective effect against Ang II-induced myocardial hypertrophy in vivo. Further, muscone and valsartan are comparable in terms of improving cardiac function and left ventricular structure, the latter being commonly used in patients with myocardial hypertrophy.

### Muscone improves Ang II-induced inflammatory response and cardiac fibrosis

**Fig. 2:**
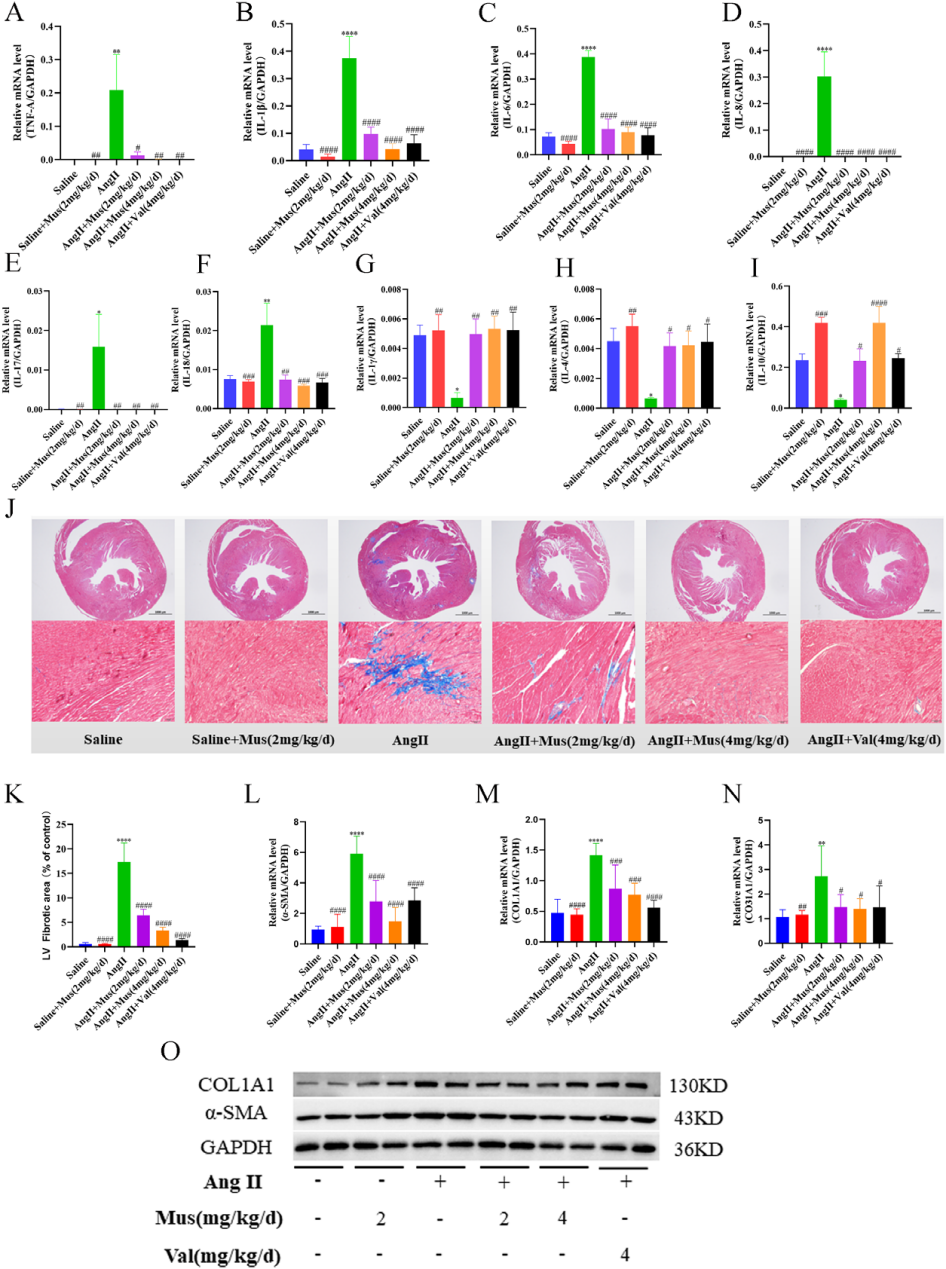
Muscone improves Ang II-induced inflammatory response and cardiac fibrosis. (**A**–**I**) The mRNA expression of TNF-α, IL-1β, IL-6, IL-17, IL-18, IL-1γ, IL-4 and IL-10 was examined by qRT-PCR. (**J**) Hypertrophic hearts stained with Masson’s trichrome. The myocardium was dyed red, while collagen fibers were coloured blue. (Scale bar = 1000 or 50 μm) The lower six photographs matched the upper six images. (**K**) Fibrotic areas were quantified in the hearts of mice using ImageJ software. (**L**–**N**) The mRNA expression of α-SMA, COL1A1, and COL3A1 was examined by qRT-PCR. (**O**) Western blot analysis showing α-SMA and COL1A1 protein levels in mouse cardiac tissues. GAPDH was used as a loading control. Data are presented as the mean ± SD (n = 3–6). *p < 0.05, **p < 0.01, ***p < 0.001, and ****p < 0.0001 vs. the saline group; #p < 0.05, ##p < 0.01, ###p < 0.001, and ####p < 0.0001 vs. the Ang II group

Increased secretion of proinflammatory cytokines in myocardial tissue is one of the characteristics of pathological cardiac hypertrophy [21]. Therefore, to investigate the anti-inflammatory effect of muscone on Ang II-induced cardiac hypertrophy, we performed qRT-PCR analysis of RNA extracted from mouse heart tissue. The mRNA expression of tumor necrosis factor-α (TNF-α), interleukin (IL)-1β, IL-6, IL-8, IL-17 and IL-18 was significantly decreased in the muscone treatment group compared to the Ang II group (Fig. 2A-F). Chemotactic cytokines such as C-C motif chemokine ligand 2(CCL2) also showed similar results (Supplementary Figure S2A). However, the expression of IL-1γ, IL-4 and IL-10 were reduced in the Ang II group compared to the saline group, but it was increased after muscone treatment (Fig. 2G-I).

Cardiac hypertrophy often causes myocardial remodeling, accompanied by hypertrophy, fibrosis, and death of cardiomyocytes [22]. Thus, we evaluated the extent of myocardial fibrosis by staining tissue sections. Paraffin-embedded sections were stained with Masson’s trichrome staining for mouse ventricles, which stained collagen fibers blue and myocardium red under normal conditions. Significant cardiac fibrosis was observed in the Ang II group, which was indicated by an increase in the area of blue staining (Fig. 2J). In the muscone treatment group, cardiac fibrosis was reduced in a concentration-dependent manner compared to Ang II-treated mice (Fig. 2K). In addition, we examined the expression of fibrosis marker genes. The mRNA expression levels of collagen (COL1A1 and COL3A1) and α-SMA, which are markers of myocardial fibrosis, were significantly elevated in the Ang II group compared to the saline group, but muscone treatment significantly suppressed the elevated mRNA levels of α-SMA, COL1A1, and COL3A1 (Fig. 2L-N). Similarly, Western blot analysis confirmed that the protein levels of COL1A1 and α-SMA were significantly lower in the muscone treatment group compared to the Ang II group (Fig. 2O, Supplementary Figure S2B, C).

Thus, these results suggested that Ang II stimulation significantly increases inflammation and cardiac fibrosis but that muscone treatment suppresses the Ang II-induced effects.

### Network pharmacological analysis and molecular docking

**Fig. 3:**
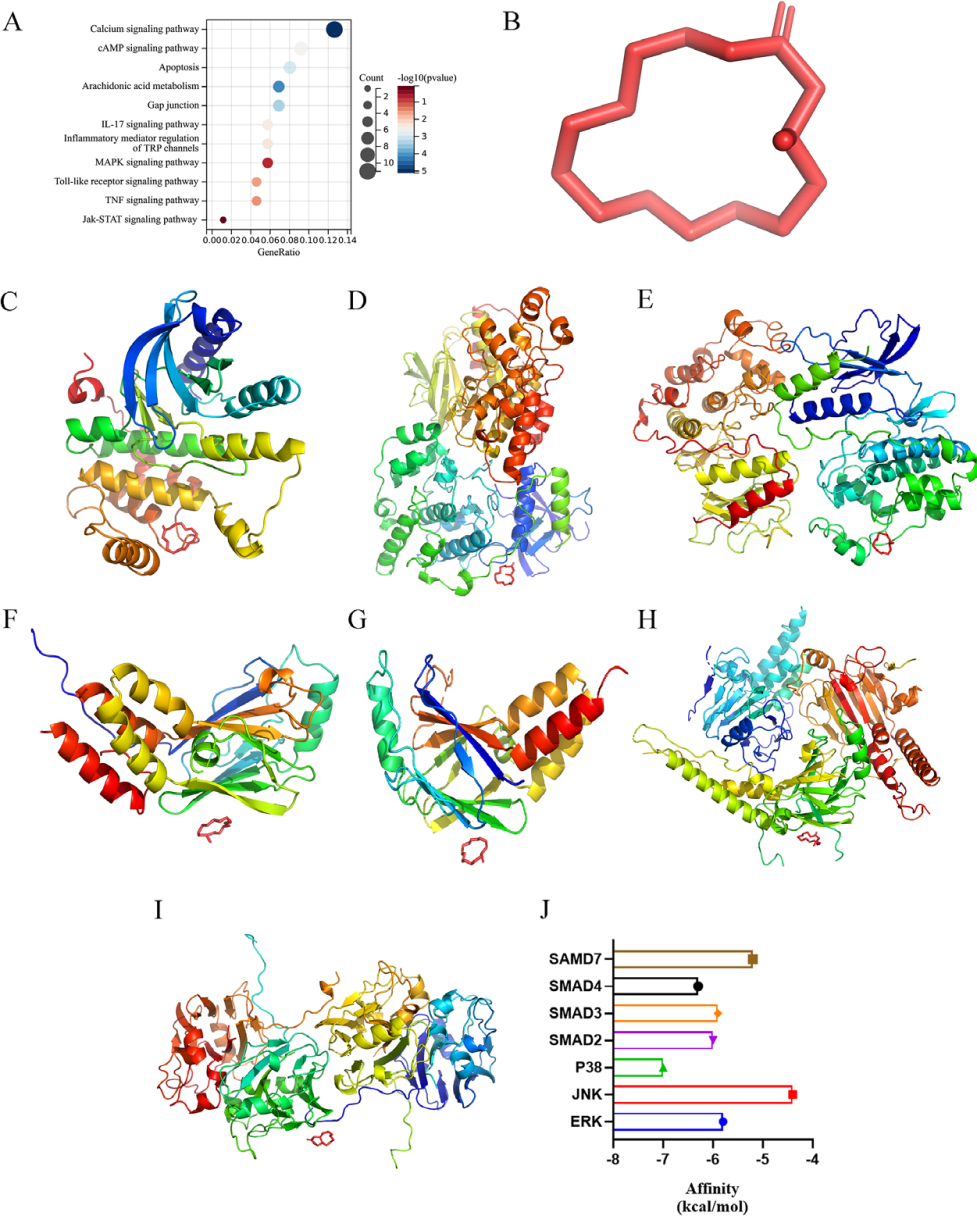
Network pharmacological analysis and molecular docking. (**A**) KEGG enrichment analysis of the potential binding target proteins. (**B**) Molecular 3D structure of muscone. (**C**-**I**) For the 3D structures of the individual protein molecules, the muscone molecule is shown in red, and the other molecules (ERK, JNK, P38, SMAD2, SMAD3, SMAD4, and SMAD7) are shown in different colors. (**J**) The bar chart shows the affinity of muscone for each molecule

To investigate the mechanism of muscone against cardiac hypertrophy, we imported the chemical structure of muscone into SwissADME for analysis and calculated the potential binding target proteins. We then performed Kyoto Encyclopedia of Genes and Genomes (KEGG) and Gene Ontology (GO) enrichment analyses of the target proteins using the clusterProfiler package in R (version 3.14.3) (Supplementary Table 2). The predicted target proteins were found to be enriched in the calcium signaling pathway, cAMP signaling pathway, apoptosis, arachidonic acid metabolism, gap junction, IL-17 signaling pathway, inflammatory mediator regulation of TRP channels, MAPK signaling pathway, Toll-like receptor signaling pathway, TNF signaling pathway, and JAK-STAT signaling pathway (Fig. 3A). GO enrichment analysis indicated that the predicted target proteins were enriched in the following biological process terms: oxygen containing compound, homeostatic process, cell-cell signaling, chemical homeostasis, response to nitrogen compound, response to organic cyclic compound, synaptic signaling, circulatory system process, and regulation of hormone levels (Supplementary Figure S3A). GO enrichment analysis indicated that the predicted target proteins were enriched in the following cellular component terms: intrinsic component of plasma membrane, synapse, neuron projection, plasma membrane region, somatodendritic compartment, synaptic membrane, presynaptic membrane, intrinsic component of presynaptic membrane, and endolysosome lumen (Supplementary Figure S3B). GO enrichment analysis indicated that the predicted target proteins were enriched in the following molecular function terms: molecular transducer activity, G protein coupled receptor activity, oxidoreductase activity, neurotransmitter receptor activity, G protein coupled amine receptor activity, hormone binding, serotonin receptor activity, steroid dehydrogenase activity, and carbonate dehydratase activity (Supplementary Figure S3C).

We then focused on the MAPK, JAK2/STAT3, and SMAD pathways. We downloaded the chemical structure of muscone from ChemSpider and converted it into a three-dimensional (3D) structure using Openbabel software (Fig. 3B). We also downloaded the protein structures of ERK, JNK, P38, SMAD2, SMAD3, SMAD4, and SMAD7 from the RCSB website, processed them by PYMOL software, and then performed molecular docking with muscone in AUTODOCK software (Fig. 3C-I). Muscone had some affinity with all of the above molecules, and among these docked molecules, P38 had the strongest affinity for muscone (Fig. 3J).

These results suggested that the potential targets of muscone may include the MAPK, JAK2/STAT3, and SMAD pathways, further validating that muscone may interact with these molecules at the charge and molecular interaction levels through molecular docking.

### Muscone inhibits Ang II-induced activation of the TGF-β/SMAD, STAT3, and MAPK signaling pathways

**Fig. 4:**
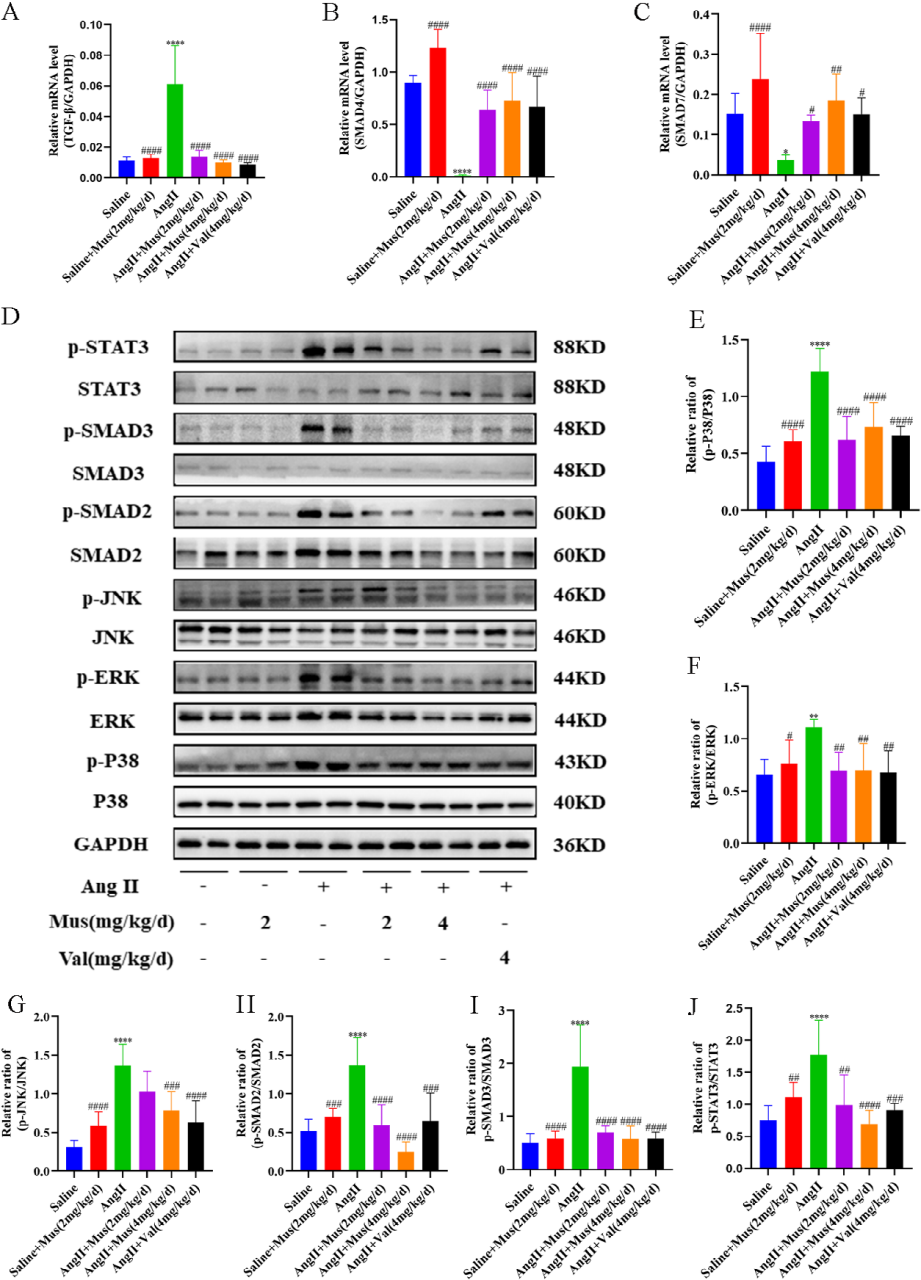
Muscone inhibits Ang II-induced activation of the TGF-β/SMAD, STAT3, and MAPK signaling pathways. (**A**-**C**) The mRNA expression of TGF-β, SMAD4, and SMAD7 was examined by qRT-PCR. (**D**) Western blot analysis showing the protein levels in mouse cardiac tissues. (**E**-**J**) Quantification of the relative changes in phosphorylation of P38, ERK, JNK, SMAD2, SMAD3 and STAT3. GAPDH was used as a loading control. Data are presented as the mean ± SD (n = 3–6). *p < 0.05, **p < 0.01, ***p < 0.001, and ****p < 0.0001 vs. the saline group; #p < 0.05, ##p < 0.01, ###p < 0.001, and ####p < 0.0001 vs. the Ang II group

Because some interaction of muscone with the above molecules was detected by molecular docking analysis, we examined the expression of these molecules in mouse hearts at the molecular level to further explore whether muscone acts through these molecules to affect Ang II-induced cardiac hypertrophy. We examined the effects of muscone on the TGF-β/SMAD, STAT3 and MAPK signaling pathways involved in Ang II-induced cardiac hypertrophy and inflammation. The mRNA level of TGF-β was increased after Ang II stimulation, but these levels were significantly decreased after muscone treatment (Fig. 4A). The mRNA levels of SMAD4 and SMAD7 were decreased after Ang II stimulation, but these levels were significantly increased after muscone treatment (Fig. 4B-C). The protein expression levels of phosphorylated STAT3, phosphorylated SMAD3, phosphorylated SMAD2, phosphorylated JNK, phosphorylated ERK, and phosphorylated P38 were significantly increased in cardiac tissue in the Ang II group. However, this expression was significantly reduced after muscone treatment (Fig. 4D). Moreover, the ratios of p-STAT3/STAT3, p-SMAD3/SMAD3, p-SMAD2/SMAD2, p-JNK/JNK, p-ERK/ERK, and p-P38/P38 were reduced in the muscone treatment group compared to the angiotensin II group (Fig. 4E-J).

Taken together, these findings suggested that muscone may inhibit Ang II-induced activation of the TGF-β/SMAD, STAT3, and MAPK signaling pathways by inhibiting phosphorylation of key proteins.

### Muscone has no significant toxic side effects

**Fig. 5:**
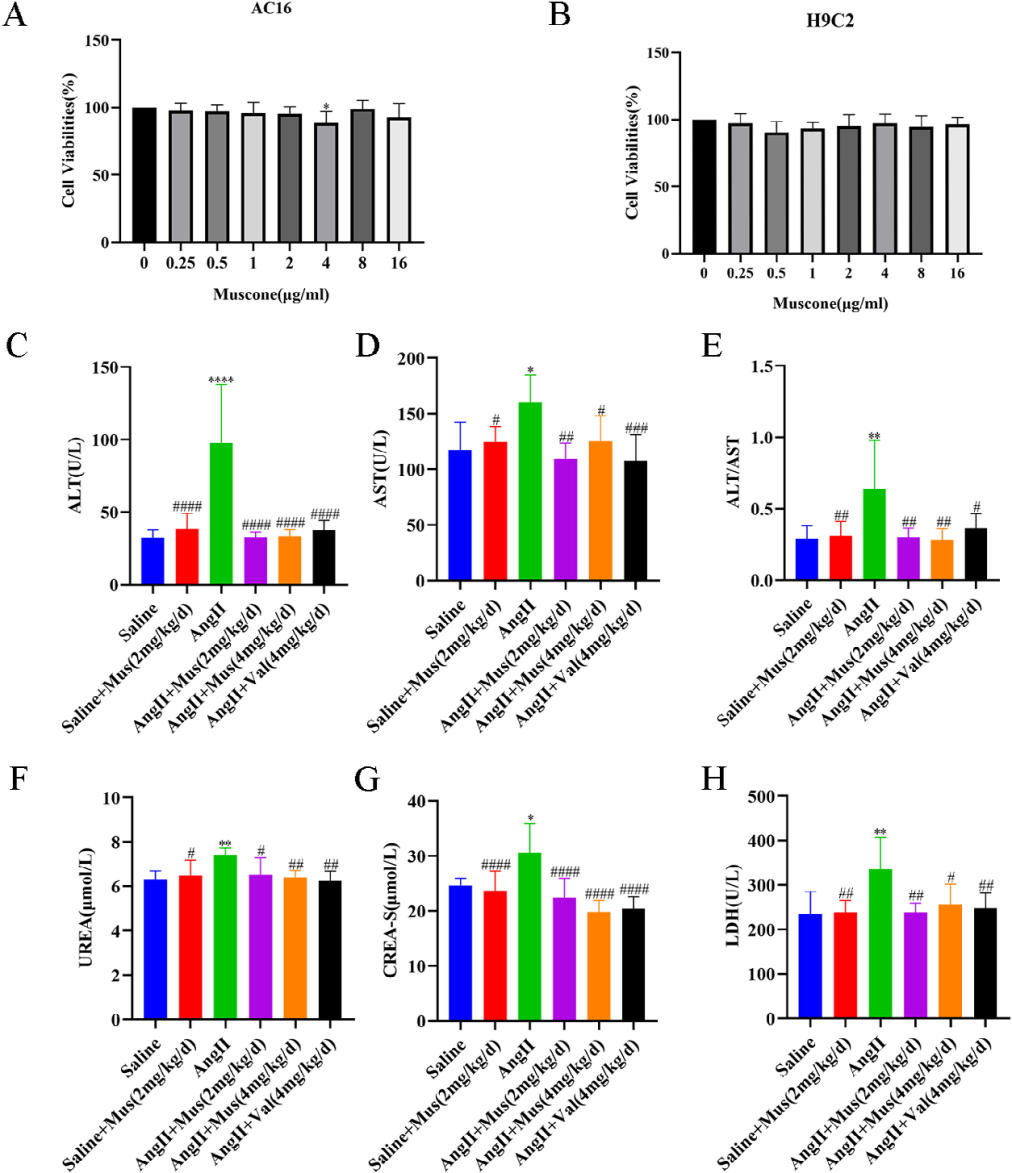
Muscone has no significant toxic side effects. (**A**, **B**) H9C2 and AC16 cells were treated with different concentrations of muscone (0–16 µg/ml) for 24 h. The cell viabilities were measured by a CCK-8 assay. (**C**-**H**) The levels of ALT, AST, ALT/AST, UREA, CREA-S, and LDH were measured in the serum samples of various mouse groups. Data are presented as the mean ± SD. *p < 0.05, **p < 0.01, ***p < 0.001, and ****p < 0.0001 vs. the saline group; #p < 0.05, ##p < 0.01, ###p < 0.001, and ####p < 0.0001 vs. the Ang II group

We next explored the effects of muscone on normal organs as unexpected adverse reactions are a major obstacle for clinical use. Some common therapeutic drugs have side effects, such as cardiotoxicity, hepatotoxicity, and nephrotoxicity, thus limiting their use. Compared to the saline group, the toxicity of the saline + muscone group (negative control) was not statistically different. We also examined the effect of muscone on the viability of H9C2 and AC16 cells using a CCK-8 assay (Fig. 5A, B), which showed no significant toxicity for muscone in these cells. Next, we examined the toxicity or adverse effects of muscone by measuring the ALT, AST, UREA, CREA-S, and LDH biochemical indexes in the serum samples from mice in each group. The levels of these biochemical parameters were increased in the Ang II group compared to the saline group but were improved or significantly reduced by muscone treatment (Fig. 5C-H). These results indicated that muscone does not damage and may even have a protective effect on the mouse heart, liver, and kidney. Thus, these findings suggested that muscone has no significant toxicity or side effects on normal organs.

## Discussion

**Fig. 6:**
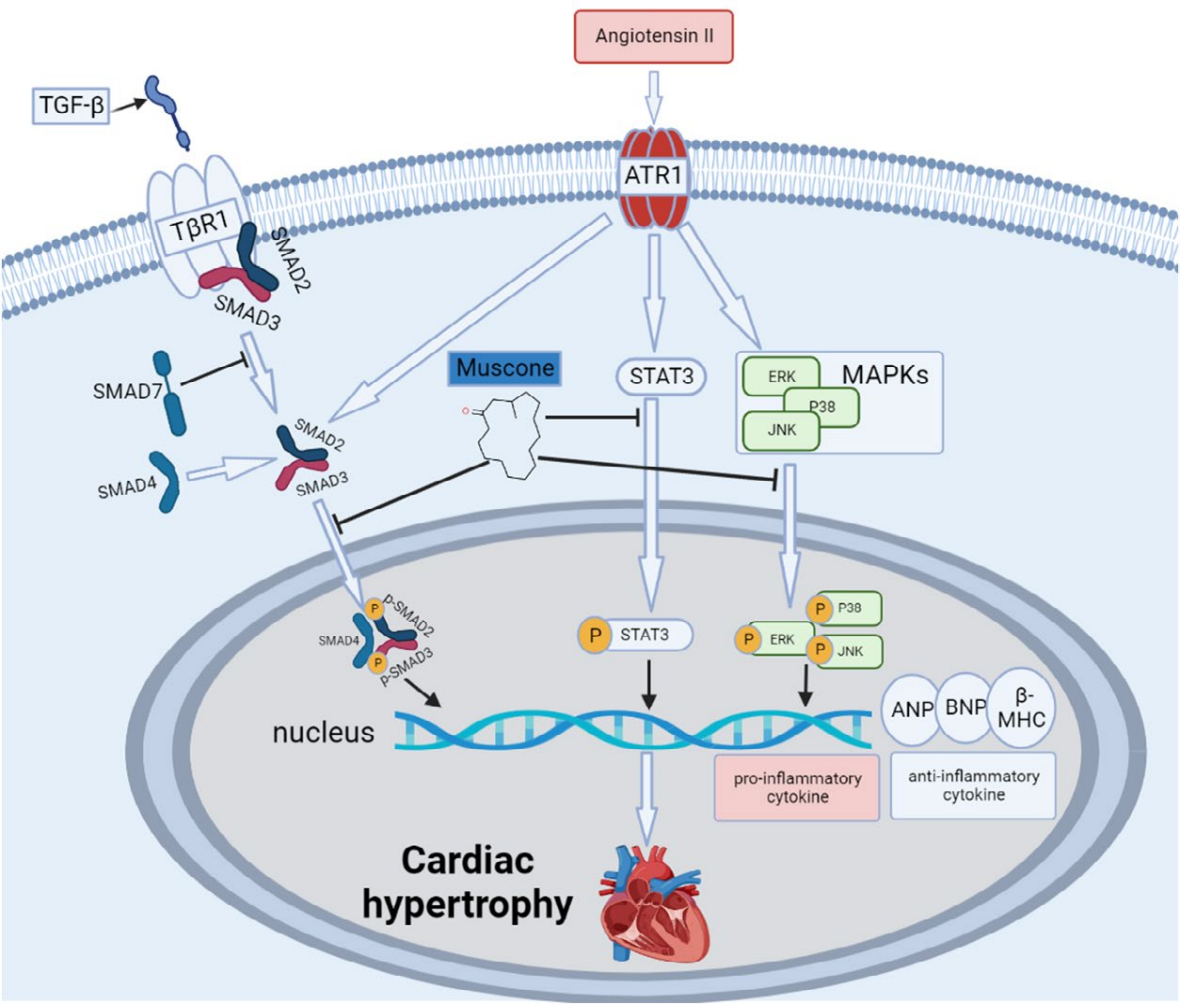
Schematic diagram of the roles of muscone in Ang II-induced cardiac hypertrophy. Ang II induced cardiac hypertrophy in vivo, which was mainly mediated through the angiotensin II type 1 receptor (AT1R). Ang II stimulation increased the secretion of proinflammatory cytokines but decreased the secretion of anti-inflammatory cytokines. At the same time, Ang II stimulation increased the expression of ANP, BNP, and β-MHC. Muscone ameliorated Ang II-provoked cardiac hypertrophy, inflammation and cardiac damage through suppressing the TGF-β/SMAD, STAT3, and MAPK signaling pathways

In the present study, we demonstrated for the first time that muscone inhibits Ang II-induced myocardial hypertrophy and myocardial injury in vivo. The present results showed that muscones significantly attenuated Ang II-induced myocardial fibrosis, remodeling, and inflammation. Mechanistically, the MAPK, STAT3, and TGF-β/SMAD pathways are closely associated with muscone cardiac protection, and muscone has no significant toxicity or side effects in vivo or in vitro (Fig. 6). In conclusion, the present study suggested that muscone may be a promising candidate for the treatment of cardiac hypertrophy and heart failure.

In pathological conditions, myocardial hypertrophy is harmful to the organism and is a key risk factor for the progression of heart failure, often leading to cardiac injury, irregular heartbeat rhythm, angina, and heart attack, and the risk of death is also severely increased with myocardial hypertrophy [23, 24]. Despite many reports describing the pathophysiological mechanisms of myocardial hypertrophy as well as cutting-edge studies, the clinical outcome of myocardial hypertrophy remains poor. Therefore, the development of new drugs to prevent and treat myocardial hypertrophy is urgent. In recent years, many researchers have extensively studied compound Chinese medicines and natural products derived from Chinese medicines, and their cardioprotective effects have been explored and applied in the clinic [25–27]. As a monomeric compound, muscone is the main component of Heart-protecting Musk Pill (HMP) [28]. There is growing evidence that muscone exerts significant beneficial effects in the cardiovascular setting. For example, muscone attenuates myocardial ischemia-reperfusion injury by inhibiting oxidative stress and enhancing SIRT3 [29]. Hong et al. found that muscone inhibits the excessive inflammatory response in myocardial infarction by targeting TREM-1. Yingqiang et al. found that inhibition of NF-κB and NLRP3 inflammasomes attenuates chronic inflammation mediated by cardiac macrophages and improves cardiac function in mice after myocardial infarction [30, 31]. However, the studies addressing the mechanism of action of muscone in relation to myocardial hypertrophy have not investigated whether muscone has a therapeutic effect on cardiovascular disease. In the present study, we investigated the relationship between muscone and myocardial hypertrophy on the basis of cellular and animal models. For a positive control, we used valsartan, an angiotensin II receptor blocker (ARBs), which has been clinically used to treat hypertension. Nordén and Burke et al. found that valsartan reduces cardiac fibrosis during left ventricular pressure overload by restoring protein kinase G (PKG) signaling in cardiac fibroblasts, and they reported that valsartan improves cardiac hypertrophy and preserves diastolic during cardiac pressure overload [32, 33]. Therefore, we evaluated the therapeutic effect of muscone in this model of cardiac hypertrophy, using valsartan as a positive control. We found that muscone inhibited Ang II-induced myocardial hypertrophy in vivo, and the therapeutic value of high-dose muscone was comparable to that of valsartan.

Myocardial fibrosis is a phenomenon of collagen accumulation within the myocardium, and it is usually characterized by elevated collagen levels [3]. In the presence of excessive left ventricular pressure, myocardial fibrosis occurs, initially as an adaptive response that maintains the firing capacity, or active (contractile) stiffness, of the hypertrophied myocardium. In the later stages of hypertrophy, myocardial interstitial fibrosis has a detrimental effect on diastolic and systolic stiffness of the myocardium, which may lead to pathological hypertrophy and heart failure. In the present study, we found that muscone significantly inhibited Ang II-induced myocardial fibrosis. Moreover, muscone effectively reduced the expression of COL1A1 and COL3A1 in vivo, and we found that the SMAD pathway may play a key role in this process. These results suggested that muscone significantly ameliorates Ang II-induced myocardial fibrosis in mice.

Proinflammatory cytokines are small molecule proteins involved in signaling pathways that have been shown to be involved in cardiovascular disease, and they have also been shown to be involved in the transition from adaptive to pathological cardiac hypertrophy during cardiac remodeling [21, 34]. Previous studies have shown that proinflammatory cytokines, such as TNF-α, IL-1β, and IL-6, are closely associated with myocardial fibrosis, pathological cardiac remodeling, and myocardial hypertrophy [12, 35]. In the present study, we examined the expression levels of the proinflammatory and anti-inflammatory cytokines in mice. The proinflammatory cytokines were significantly lower after muscone treatment compared to the Ang II group alone, and opposite results were observed for the anti-inflammatory cytokines. The regulation of inflammatory responses and myocardial hypertrophy by Ang II via the MAPK and SMAD signaling pathways has been extensively studied [36–39]. In addition, IL-6, which is produced by cardiomyocytes in response to hypertrophic stimuli, activates the STAT3 and MAPK signaling pathway in myocardial hypertrophy [40, 41]. Muscone has been shown to improve diabetic peripheral neuropathy by activating the AKT/mTOR signaling pathway [42]. Yu et al. found that muscone alleviates inflammatory pain by inhibiting the NOX4/JAK2-STAT3 pathway and NLRP3 inflammasomes via microglia activation-mediated inflammatory responses [43]. Qian et al. (2010) reported that muscone inhibits the expression of prostaglandin E2, 6-keto-prostaglandin F1 alpha, IL-1β, and TNF-α as well as restores the structural deformation of degenerative intervertebral discs [44]. In addition, Liu et al. (2022) reported that muscone exerts antidepressant-like effects by reducing neuroinflammation and oxidative stress in a mouse model of chronic restraint stress [45]. In the present study, molecular docking predicted that muscone may function in the heart through binding molecules in the MAPK and SMAD pathway. The present experiments clearly demonstrated that muscone significantly reduced the expression of p-ERK, p-JNK, p-P38, p-SMAD2, and p-SMAD3 compared to Ang II treatment alone. These results suggested that muscone attenuates ANG II-induced myocardial hypertrophy, at least in part, by inhibiting the MAPK and TGF-β/SMAD signaling pathways. However, more detailed studies are needed in the future to determine the exact molecular pathways underlying the anti-hypertrophic effects of muscone.

As we mentioned previously, several studies have shown that muscone is protective against certain diseases. For example, muscone ameliorates lipopolysaccharide (LPS)-induced depressive-like behaviors and inhibits neuroinflammation in the prefrontal cortex of mice [46]. In addition, muscone suppresses inflammatory responses and neuronal damage in a rat model of cervical spondylotic myelopathy by modulating Drp1-dependent mitochondrial fission [47]. These studies imply that muscone has the potential to be developed as a clinical agent to alleviate neuroinflammation. Therefore, studies on the toxicity and adverse effects of muscone are particularly important. In this regard, we conducted a study on the toxicity of muscone to the body by using a muscone treatment group as a negative control to test liver and kidney functions as well as cell viability using a CCK-8 assay. The results showed that muscone had no significant toxicity or adverse effects on normal organs, such as the heart, liver, and kidneys.

## Conclusions

In conclusion, the present study demonstrated for the first time that muscone significantly inhibits Ang II-mediated myocardial hypertrophy in mice, and it predicted for the first time the affinity and potential binding sites of muscone with P38, ERK, and JNK in the MAPK pathway as well as SMAD2, SMAD3, SMAD4, and SMAD7 in the TGF-β/SMAD pathway by molecular docking analysis. We also verified that muscone reduces myocardial fibrosis, decreases proinflammatory cytokine secretion, and inhibits, thus exerting cardioprotective effects through the activation of the STAT3, TGF-β/SMAD, and MAPK signaling pathways. Finally, we demonstrated that muscone has no significant toxicity or side effects on normal cells and organs. Therefore, the muscone natural product may be a new and effective drug for the treatment of cardiac hypertrophy and heart failure.

## Electronic supplementary material

Below is the link to the electronic supplementary material.

Supplementary Material 1

Supplementary Material 2: List of primers

Supplementary Material 3: Predicted results for potential target proteins

## Data Availability

The data are available from the corresponding author or first author upon reasonable request.
