# Supplementary material for: Muscone inhibits angiotensin II–induced cardiac hypertrophy through the STAT3, MAPK and TGF-β/SMAD signaling pathways
Source: Mol Biol Rep. 2023 Dec 29;51(1):39. doi: 10.1007/s11033-023-08916-1 (PMC10756871; doi:10.1007/s11033-023-08916-1)

**Supplementary Figure S1 |** (A-F) Statistical results for the LVIDs, LVM, HR, IVSd, IVSs, and LVIDd parameters. (G, H) Quantification of ANP and β-MHC protein levels in different groups. GAPDH was used as a loading control. Data are presented as the mean ± SD. *p < 0.05, **p < 0.01, ***p < 0.001, and ****p < 0.0001 vs. the saline group; #p < 0.05, ##p < 0.01, ###p < 0.001, and ####p < 0.0001 vs. the Ang II group.


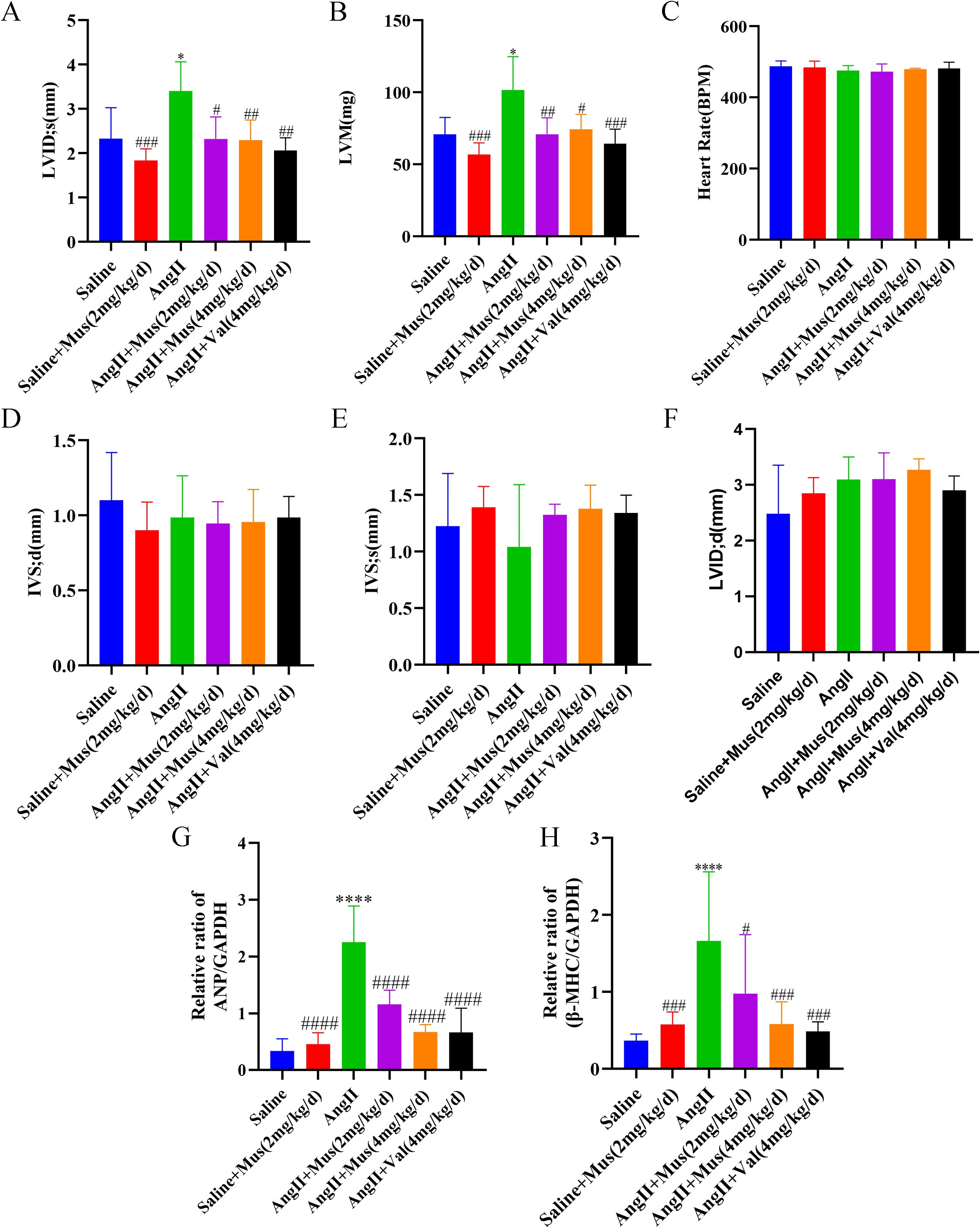


**Supplementary Figure S2 |**(A) qRT-PCR was used to analyze the mRNA expression of CCL2. (B, C) Quantification of α-SMA and COL1A1 protein levels in different groups. GAPDH was used as a loading control. Data are presented as the mean ± SD. *p < 0.05, **p < 0.01, ***p < 0.001, and ****p < 0.0001 vs. the saline group; #p < 0.05, ##p < 0.01, ###p < 0.001, and ####p < 0.0001 vs, the Ang II group.


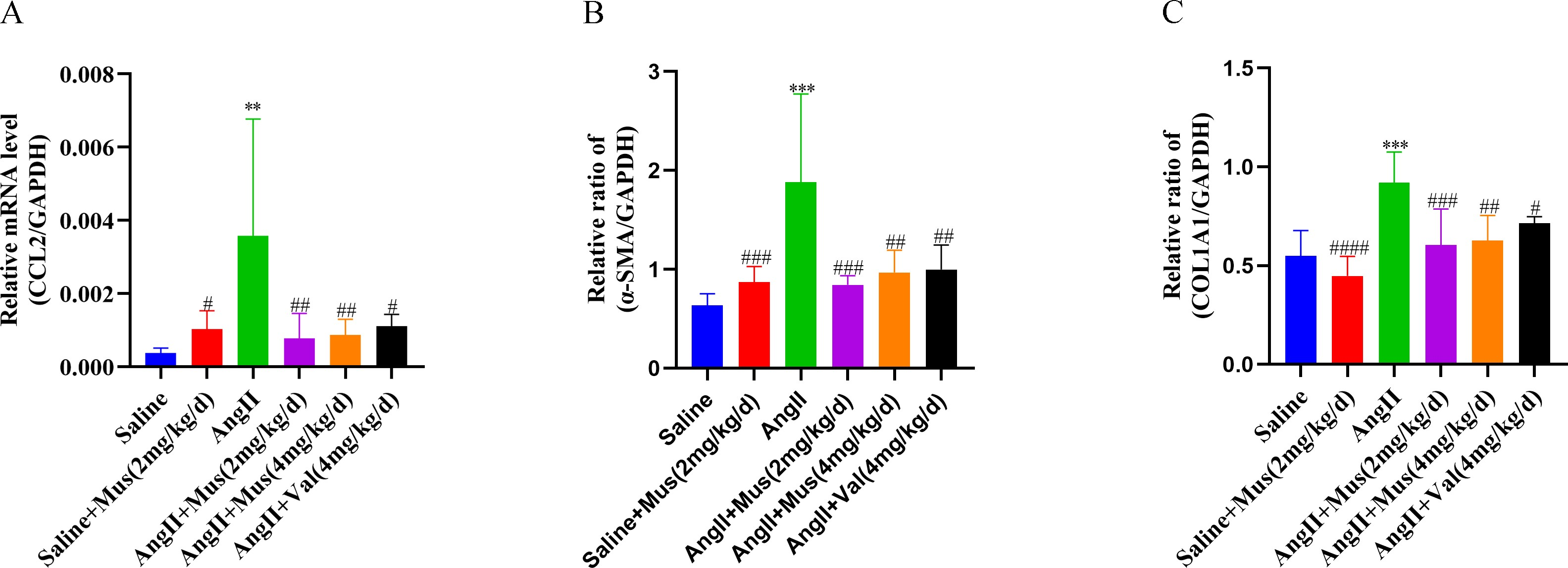


**Supplementary Figure S3 |** (A-C) The bubble diagram represents the biological process, cellular component, and molecular function terms of the target proteins in the GO enrichment analysis.


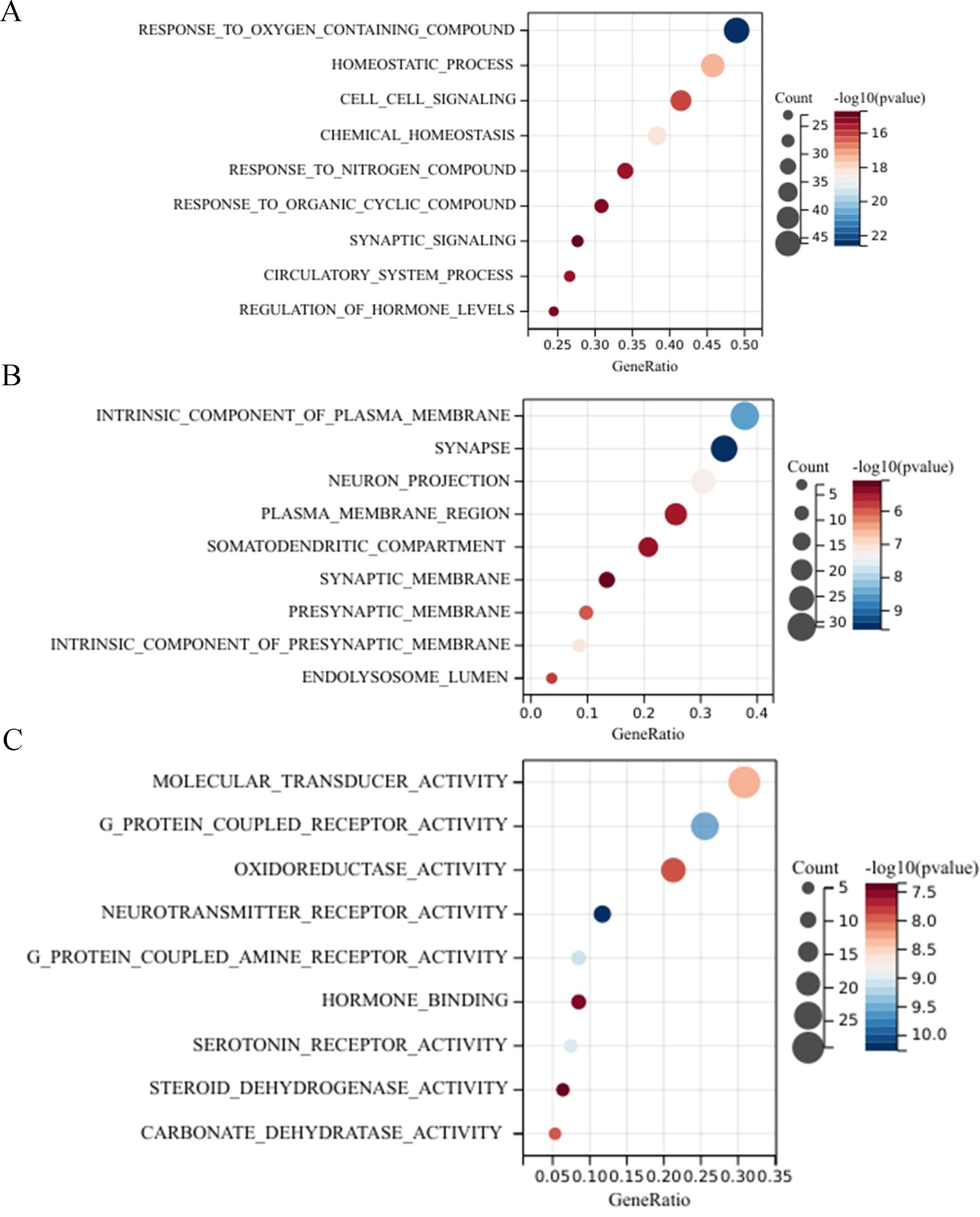

Supplement: Supplementary file 1 — Supplementary Material 1 [file 11033_2023_8916_MOESM1_ESM.docx]
