# Supplementary material for: Muscone inhibits angiotensin II–induced cardiac hypertrophy through the STAT3, MAPK and TGF-β/SMAD signaling pathways
Source: Mol Biol Rep. 2023 Dec 29;51(1):39. doi: 10.1007/s11033-023-08916-1 (PMC10756871; doi:10.1007/s11033-023-08916-1)
Supplement: Supplementary file 2 — Supplementary Material 2: List of primers [file 11033_2023_8916_MOESM2_ESM.docx]

Supplementary table.1. List of primers

| Target gene |  | Sequences | Target gene |  | Sequences |
| --- | --- | --- | --- | --- | --- |
| Mouse GAPDH | Forward | 5’-GGTTGTCTCCTGCGACTTCA-3’ | Mouse CCL2 | Forward | 5’- TTAAAAACCTGGATCGGAACCAA-3’ |
|  | Reverse | 5’-GGTGGTCCAGGGTTTCTTACTC-3’ |  | Reverse | 5’- GCATTAGCTTCAGATTTACGGGT -3’ |
| Mouse ANP | Forward | 5’-CACAGATCTGATGGATTTCAAGA-3’ | Mouse α-MHC | Forward | 5’- GTCCAAGTTOCGCAAGGT -3’ |
|  | Reverse | 5’-CCTCATCTTCTACCGGCATC-3’ |  | Reverse | 5’- CAAGGCTGGTCCATGCTCC -3’ |
| Mouse BNP | Forward | 5’-GAAGGTGCTGTCCCAGATGA-3’ | Mouse(il-8)Cxcl15 | Forward | 5’-TGCTATCACTTCCTTTCTGTTGC-3’ |
|  | Reverse | 5’-CCAGCAGCTGCATCTTGAAT-3’ |  | Reverse | 5’-GTGGATTGTTCTGGAGACTG-3’ |
| Mouse IL-1β | Forward | 5’-TCGCAGCAGCACATCAACAAGAG-3’ | Mouse IL-17 | Forward | 5’-TTTAACTCCCTTGGCGCAAAA-3’ |
|  | Reverse | 5’-AGGTCCACGGGAAAGACACAGG-3’ |  | Reverse | 5’-CTTTCCCTCCGCATTGACAC-3’ |
| Mouse TNF-a | Forward | 5’-GCGACGTGGAACTGGCAGAAG-3’ | Mouse IL-4 | Forward | 5’-GGTCTCAACCCCCAGCTAGT-3’ |
|  | Reverse | 5’-GCCACAAGCAGGAATGAGAAGAGG-3’ |  | Reverse | 5’-GCCGATGATCTCTCTCAAGTGAT-3’ |
| Mouse IL-6 | Forward | 5’-CTTCTTGGGACTGATGCTGGTGAC-3’ | Mouse IL-1R1 | Forward | 5’-GTGCTACTGGGGCTCATTTGT-3’ |
|  | Reverse | 5’-AGGTCTGTTGGGAGTGGTATCCTC-3’ |  | Reverse | 5’-GGAGTAAGAGGACACTTGCGAAT-3’ |
| Mouse myh7 | Forward | 5’-AGCTCCTGGAAAGAAACACTAA-3’ | Mouse IL-10 | Forward | 5’-GCTCTTACTGACTGGCATGAG-3’ |
|  | Reverse | 5’-GTAACAGTACTTGGCATACGTG-3’ |  | Reverse | 5’-CGCAGCTCTAGGAGCATGTG-3’ |
| Mouse COL1A1 | Forward | 5’-GCTCCTCTTAGGGGCCACT-3’ | Mouse IL18 | Forward | 5’-TCCAACTGCAGACTGGCAC-3’ |
|  | Reverse | 5’-CCACGTCTCACCATTGGGG-3’ |  | Reverse | 5’-GGCAGGAGTCCAGAAAGCAT-3’ |
| Mouse COL3A1 | Forward | 5’-CTGTAACATGGAAACTGGGGAAA-3’ | Mouse SMAD4 | Forward | 5’-GCATCGACAGAGACATACAG-3’ |
|  | Reverse | 5’-CCATAGCTGAACTGAAAACCACC-3’ |  | Reverse | 5’-CAACAGTAACAATAGGGCAG-3’ |
| Mouse SMAD7 | Forward | 5’-GAATCTTACGGGAAGATCAACCC-3’ |  |  |  |
|  | Reverse | 5’-CGCAGAGTCGGCTAAGGTG-3’ |  |  |  |
